# Correspondence on Lovell et al.: identification of chicken genes previously assumed to be evolutionarily lost

**DOI:** 10.1186/s13059-017-1231-1

**Published:** 2017-06-14

**Authors:** Susanne Bornelöv, Eyal Seroussi, Sara Yosefi, Ken Pendavis, Shane C. Burgess, Manfred Grabherr, Miriam Friedman-Einat, Leif Andersson

**Affiliations:** 1 Science for Life Laboratory, Department of Medical Biochemistry and Microbiology, Uppsala University, Uppsala, SE-751 23 Sweden; 2 Present Address: Wellcome Trust Medical Research Council Stem Cell Institute, University of Cambridge, Cambridge, CB2 1QR UK; 3 Agricultural Research Organization, Volcani Center, Rishon LeZion, Israel; 4 College of Agriculture and Life Sciences, University of Arizona, Tucson, AZ 85721-0036 USA; 5 Bioinformatics Infrastructure for Life Sciences, Uppsala University, Uppsala, Sweden; 6 Department of Animal Breeding and Genetics, Swedish University of Agricultural Sciences, Uppsala, SE-750 07 Sweden; 7 Department of Veterinary Integrative Biosciences, College of Veterinary Medicine and Biomedical Sciences, Texas A&M University, College Station, TX 77843-4458 USA

## Abstract

Through RNA-Seq analyses, we identified 137 genes that are missing in chicken, including the long-sought-after nephrin and tumor necrosis factor genes. These genes tended to cluster in GC-rich regions that have poor coverage in genome sequence databases. Hence, the occurrence of syntenic groups of vertebrate genes that have not been observed in Aves does not prove the evolutionary loss of such genes.

Please see related Research article: http://dx.doi.org/10.1186/s13059-014-0565-1 and Please see response from Lovell et al: https://www.dx.doi.org/10.1186/s13059-017-1234-y

**Electronic supplementary material:**

The online version of this article (doi:10.1186/s13059-017-1231-1) contains supplementary material, which is available to authorized users.

A recent paper reported that 274 protein-encoding genes were missing from sequencing data from 60 bird species [1]. Most of them were organized in conserved syntenic clusters in non-avian vertebrates, suggesting that their loss in the avian lineage had occurred through genomic deletions of gene blocks. This hypothesis was supported by another study reporting that 640 protein-encoding genes were missing from 48 bird genomes [2]; the authors of this second study made a similar suggestion that large segmentally deleted regions had been lost during microchromosome evolution in birds. However, our recent discovery of leptin genes with ~70% GC content in chicken and duck [3], and the new identification of 89 GC-rich genes [4], suggested an alternative hypothesis of a technical barrier to explain the ‘missing genes’. To further explore this, RNA-Seq data from visceral fat, hypothalamus, and pituitary tissues from two types of chickens, broilers and layers (Additional file 1: Table S1), were used for de novo transcriptome assembly and identification of novel genes.

The initial set of 588,683 transcripts obtained using Trinity [5] was reduced to 257,700 after removing transcripts that were expressed at low levels. We mapped the transcripts to the chicken reference genome build consistent with the previous studies [1, 2] using Blat and Blast, and retained 8395 sequences without alignments. These transcripts were then characterized on the basis of sequence similarity to known genes in other vertebrates using the Trinotate pipeline (https://trinotate.github.io), which searches for sequences encoding known protein domains, transmembrane domains, and signal peptides (Additional file 1: Tables S2 and S3a). Genes that were already known in chicken were removed by comparing their gene symbols with those in Ensembl (release 80), RefSeq, and Entrez Gene, resulting in 1878 novel gene-candidate transcripts representing 1063 genes (Additional file 1: Tables S3b and S4).

To increase specificity and to remove multiple transcript isoforms, we tested each transcript by reciprocal Blastn against the full transcriptome assembly (588,683 transcripts), and Blastx against the set of coding sequences predicted by TransDecoder (https://transdecoder.github.io), consisting of 111,457 sequences. The remaining set yielded 194 transcripts encompassing 190 distinct high-confidence genes (Additional file 1: Table S5). Through Blastn, we found that 55 loci had already been recovered as annotated genes in an updated genome build (Galgal5) released after the previous studies. In addition, 47 genes mapped to the genome but lacked annotations, while another 51 genes were annotated as uncharacterized or putative proteins (Additional file 1: Table S6). One discrepancy in annotation between our genes and Galgal5 was observed for the *RSAD1* transcript, which was annotated as *MYCBPAP* in Galgal5. Closer inspection revealed that these two genes, which are close neighbors in the human genome, have been mistakenly merged into *MYCBPAP* in Galgal5. Therefore, we considered *RSAD1* as a novel annotation (Additional file 1: Table S6).

Among the remaining 38 genes (Additional file 1: Table S6) with no sequence similarity to any genome build are the tumor necrosis factor (*TNF*) and nephrin (*NPHS1*), which have been reported as missing from birds in several studies (Table 1) but which are critically important in vertebrate biology and have extensively been studied in non-avian vertebrates (there are more than 130,000 publications in PubMed on *TNF* and 1300 on *NPHS1*). These genes were subjected to full-cDNA-sequence determination, exon characterization, RT-PCR validation, and expression profiling using RNA-Seq data from red junglefowl (Additional file 2: Figures S1 and S2; Additional file 2: Tables S9 to S12). The similarity in sequences, exon–intron junctions, and characteristic expression profiles confirmed the identification of chicken *NPHS1* and *TNF*, thus resolving the long discussion as to why these genes have been missing from the genome assembly despite their established essential biological function in other species (for examples, see [6–12]).

**Table 1:** Characterization of the novel genes reported missing in previous studies

Previously reported list	No. of missing genes	Found in our intermediate set	Found in our high-confidence list	Gene symbols
Predicted absent in birds [1]	274	36 (13%)	8 (3%)	***FLT3LG***, *LPPR2*, ***NPHS1***, *PLCB3* ^a^, *PRSS8*, ***RCN3***, *TRMT1*, *TSPAN31*
Predicted missing in chickens but not in all birds [1]^b^	336	152 (45%)	50 (15%)	*ALKBH7*, *ASB16*, *ATAT1*, *ATG4D*, *B9D2*, *CACNG7*, *CACNG8*, *CAMSAP3*, ***CARM1***, ***CCDC106***, *CCDC120*, ***CCDC22***, *CIC*, *CLASRP*, *CLPP*, *COPZ1*, *CYTH2*, *ESYT1*, *GEMIN7*, *GPKOW*, *GTF2F1*, *JOSD2*, *KRI1*, *LMTK3*, *MAP2K7*, *METTL1*, *METTL3*, ***MRPS18B***, *NDUFB7*, *PIH1D1*, *POU6F1*, *PPP1R12C*, *PPP1R18*, *PPP5C*, *PRKCSH*, *PRPF31*, ***PRR12***, *SAMD1*, ***SCAF1***, *SEMA4C*, ***SLC39A7***, *SMG9*, *SSR4*, *TFPT*, ***TRAPPC1***, *TSR2*, ***U2AF2***, *UXT*, *YIF1B*, *ZNF653*
Predicted absent in birds [2]	640	100 (16%)	29 (4.5%)	***ADAT3***, *ALKBH7*, *C11ORF95*, ***C2ORF68***, ***CCDC22***, ***CDIPT***, *CGREF1*, *CIC*, *CXXC1*, *FRMD8*, *HUWE1*, *IKBKG*, *KRI1*, *LMTK3*, *MBD1*, *MUS81*, ***NPHS1***, *OPA3*, ***PHF1***, *PIH1D1*, *PLCB3* ^a^, *PPP1R12C*, *PRKCSH*, *RCE1*, *SSSCA1*, *TFPT*, ***TNF*** ^*d*^, *UXT*, *ZNF653*
Predicted absent by both studies [1, 2]	99	7 (7%)	2 (2%)	***NPHS1*** ^c^, *PLCB3* ^a^
Lost adipokines [6]	4	1 (25%)	1 (25%)	***TNF*** ^**d**^

Eleven genes are shared between row 2 (Lovell et al. [1]) and row 3 (Zhang et al. [2]): *ALKBH7*, *CCDC22*, *CIC*, *KRI1*, *LMTK3*, *PIHID1*, *PPP1R12C*, *PRKCSH*, *TFPT*, *UXT*, and *ZNF653*

^a^ *PLCB3* was selected manually from the intermediate list of novel genes as a dropout due to misannotation of its quail (*Coturnix japonica*) ortholog (LOC107307599), demonstrating that the intermediate gene list (Additional file 1: Table S4) may contain additional novel genes

^b^Based on the genes listed in Tables S4a, S4b, S6a, and S6b in Lovell et al. [1]

^c^Also reported missing in other publications (e.g. [7, 14])

^d^Also reported missing also in Zhang et al. [2] and in additional publications (e.g. [10, 15])

(i) Bold and underlined, (ii) underlined, (iii) underlined by dashed line, and (iv) non-underlined symbols represent (i) novel sequences with no sequence similarity in any genome build, (ii) sequences present in Galgal5 but lacking annotation, (iii) sequences present in Galgal5 as uncharacterized or putative, or (iv) sequences present and annotated in Galgal5, respectively

Mass spectrometry analysis of fat tissue from the same chickens confirmed the identification of *MEPCE*, *NPC1L1*, *PHF1*, *MRPS18*, and *SF3B2* at *P* < 0.01, and the expression of *AMIGO1*, *CYAB*, *FKBP11*, *MGAT1*, *MOGS*, *MRI1*, *MTX1*, *POLR3D*, *PEA15*, and *TXNIP* at *P* < 0.05 (Additional file 1: Tables S4, S5, and S8). To further validate the novel genes in the context of species phylogeny, we selected 11 genes with complete coding sequences predicted by TransDecoder (Additional file 3: Table S13) and at least four reported orthologous protein sequences in the NCBI protein database, for analysis of protein identity with the predicted chicken amino acid sequence using pBlast. As expected, the relative degrees of sequence identity were inversely correlated with evolutionary distance for most transcripts (*r* = –1 to –0.7), with three exceptions resulting from high conservation.

Comparing these genes to the genes previously reported as missing [1, 2, 6] recovered 74 overlapping gene symbols (Table 1). A higher proportion of the genes reported missing only in chickens was identified compared to those reported missing in all avian species (15% and 3–4.5%, respectively). The recovered transcripts had very high GC content (68%; Additional file 3: Figure S3b), further supporting the hypothesis that many of the genes that are currently missing from the draft genome eluded previous identification because of their high GC content [3, 4].

When exploring the location of novel genes recovered by the updated genome build, we observed that most genes (76%) were located on unplaced scaffolds, probably representing uncharacterized microchromosomes. Among those that mapped to known chromosomes, the majority (80%) were localized to microchromosomes, which are estimated to contain 50% of protein-coding genes in chickens [13]. Surprisingly, many of the mapped genes appeared in clusters. Mapping positions of the human orthologs demonstrated that the organization of 80% of the mapped novel genes was in syntenic clusters (Table 2). The strong tendency of these novel genes to cluster indicated their location in recalcitrant chromosomal regions with high GC content, primarily on microchromosomes. The methods used in this study are detailed in Additional file 4: Detailed materials and methods.

**Table 2:** Overview of novel genes missing from the Galgal4 assembly but present in Galgal5

Trinity ID	Predicted gene	Galgal5 mapping			Human ortholog (hg38)	Cluster^a^
		Genes	Chromosome	Coordinates		
c192514_g2_i1	*RRS1*	*RRS1*	chr2	115,487,692–115,488,635	chr8:66,429,028–66,430,733	–
c144374_g1_i1	*KHK*	*KHK*	chr3	104,952,675–104,954,000	chr2:27,086,747–27,100,751	1
c150768_g1_i3	*CGREF1*	*CGREF1*	chr3	104,955,106–104,955,990	chr2:27,100,594–27,119,103	1
c191309_g1_i2	*ANKRD66*	*LOC101750448*	chr3	110,320,024–110,320,850	chr6:46,746,917–46,759,506	–
c190219_g1_i1	*ADO*	*ADO*	chr6	8,089,943–8,090,591	chr10:62,804,857–62,808,483	–
c165457_g1_i6	*ABHD14B*	*LOC107056876*	chr12random_Scaffold5645	10,835–12,580	chr3:51,968,510–51,983,409	–
c181867_g2_i3	*RSAD1*	*MYCBPAP*	chr18	10,429,164–10,430,334	chr17:50,508,384–50,531,497	–
c160691_g1_i2	*BOLA3*	*BOLA3*	chr22	2,880,009–2,880,858	chr2:74,135,398–74,147,994	2
c178063_g1_i8	*SEMA4C*	*SEMA4C*	chr22random_Scaffold1011	444–4,447	chr2:96,859,716–96,869,971	2
c156624_g2_i1	*CIART*	*CIART*	chr25	2,384,775–2,385,633	chr1:150,282,543–150,287,093	3
c165802_g2_i1	*CRTC2*	*CRTC2*	chr25	2,075,046–2,076,072	chr1:153,947,675–153,958,625	3
c189493_g2_i1	*C17orf96*	*LOC107055293*	chr27	4,355,476–4,355,902	chr17:38,671,703–38,675,421	4
c151660_g2_i1	*KRI1*	*LOC107055293*	chr27	4,357,140–4,357,428	chr19:10,553,078–10,566,037	4
c167546_g1_i3	*FBXW9*	*FBXW9*	chr30random_Scaffold7361	448–2,027	chr19:12,688,917–12,696,643	5
c160528_g1_i2	*DHPS*	*DHPS*,*WDR83*	chr30random_Scaffold7361	2,298–5,407	chr19:12,675,721–12,681,902	5
c150426_g1_i4	*YIF1B*	*YIF1B*	chr32random_Scaffold22667	160–217	chr19:38,305,118–38,315,963	6
c167964_g1_i2	*B9D2*	–	chr32random_Scaffold15198	71–292	chr19:41,354,421–41,364,173	6
c164748_g1_i1	*OPA3*	*OPA3*	chr32random_Scaffold826	46,400–48,070	chr19:45,546,281–45,584,819	6
c148689_g1_i2	*SNRPD2*	*SNRPD2*	chr32random_Scaffold19601	235–1,401	chr19:45,687,454–45,692,333	6
c163802_g1_i1	*GRASP*	*GRASP*	chr33	1,916–6,474	chr12:52,006,940–52,015,864	7
c178972_g2_i2	*ESYT1*	*ESYT1*	chr33	679,134–685,279	chr12:56,128,056–56,144,671	7
c171696_g1_i1	*APOF*	*APOF*	chr33	776,046–776,629	chr12:56,360,569–56,362,823	7
c100851_g1_i1	*HOXC4*	*HOXC4*	chr33	1,095,140–1,096,547	chr12:54,016,931–54,055,327	7
c186414_g2_i1	*COPZ1*	*COPZ1*	chr33	1,170,192–1,174,833	chr12:54,325,127–54,351,849	7
c146677_g1_i1	*DAZAP2*	–	chr33	1,573,156–1,573,299	chr12:51,238,292–51,243,933	7

^a^This column indicates clusters of neighboring genes that are largely supported by the human orthologs

## Conclusions

Our RNA-Seq study, combined with extensive bioinformatics analysis, recovered 191 novel genes that were missing from previous chicken assemblies, 38 of which are still not present in the most recent genome build (Galgal5), as well as an additional 47 that are at least partially present in Galgal5 but lacking proper annotation. The high GC content (68% on average), the microchromosomal location of the majority of the novel genes (80%) covered by Galgal5, and their high tendency to cluster into syntenic blocks (80%) suggest that the novel genes were not found in earlier analyses because of their position in GC-rich gene clusters, rather than due to chromosomal fragmentation and loss. In addition, the identification and characterization of *NPHS1* and *TNF*, which are expected to be essential for avian physiology, and which are still missing from the latest genome build, emphasizes the importance of striving towards a repertoire of known and characterized genes that is as complete as possible.

## Additional files

Additional file 1: Overview of the RNA-Seq data and filtration of the novel gene candidates. **Table S1.** Information about the RNA-Seq data. **Table S2.** The initial set of 2810 candidate novel transcripts. **Table S3.** Annotation, characterization, and filtering of the novel transcripts. **Table S4.** The intermediate set of 1878 transcripts representing 1063 candidate novel genes. **Table S5.** The high-confidence set of 194 transcripts representing 191 novel genes. **Table S6.** The 191 novel genes not included in Galgal4; 54 of these are correctly annotated while 137 are missing or lack correct annotation in Galgal5. **Table S7.** Characterization of the novel genes according to predicted cellular localization. **Table S8.** Identification of the novel genes in Galgal5 genome assembly and by Mass-Spec analysis in adipose tissue. (XLS 2362 kb)

Additional file 2: Characterization of NPHS1 and TNF. **Figure S1.** Predicted full length cDNA sequence of *NPHS1* and its characterization. **Figure S2.** Predicted full length cDNA sequence of *TNF* and its characterization. **Table S9.** Coding sequence of chicken NPHS1 and TNF predicted transcripts. **Table S10.** List of *NPHS1* and *TNF* exons in human, turtle, and chicken. **Table S11.** List of primers used for RT-PCR. **Table S12.** Probes used for expression profiling in the Sequence Read Archive (SRA) database. (PDF 1672 kb)

Additional file 3: Characterization of the high confidence novel genes. **Table S13.** Phylogenetic analysis of representative novel genes. **Figure S3.** Characterization of the novel transcripts. (PDF 232 kb)

Additional file 4: Detailed materials and methods. Animals and tissue sampling. RNA-seq. Bioinformatic analysis. RT-PCR. Mass spectrometry analysis (MS). (PDF 283 kb)

## Abbreviations

NPHS1: Nephrin
TNF: Tumor necrosis factor
