# Supplementary material for: Correspondence on Lovell et al.: identification of chicken genes previously assumed to be evolutionarily lost
Source: Genome Biol. 2017 Jun 14;18:112. doi: 10.1186/s13059-017-1231-1 (PMC5470226; doi:10.1186/s13059-017-1231-1)
Supplement: Supplementary file 2 — Characterization of NPHS1 and TNF. Figure S1. Predicted full length cDNA sequence of NPHS1 and its characterization. Figure S2. Predicted full length cDNA sequence of TNF and its characterization. Table S9. Coding sequence of chicken NPHS1 and TNF predicted transcripts. Table S10. List of NPHS1 and TNF exons in human, turtle, and chicken. Table S11. List of primers used for RT-PCR. Table S12. Probes used for expression profiling in the Sequence Read Archive (SRA) database. (PDF 1672 kb) [file 13059_2017_1231_MOESM2_ESM.pdf]

Additional file 2: Characterization of *NPHS1* and *TNF*

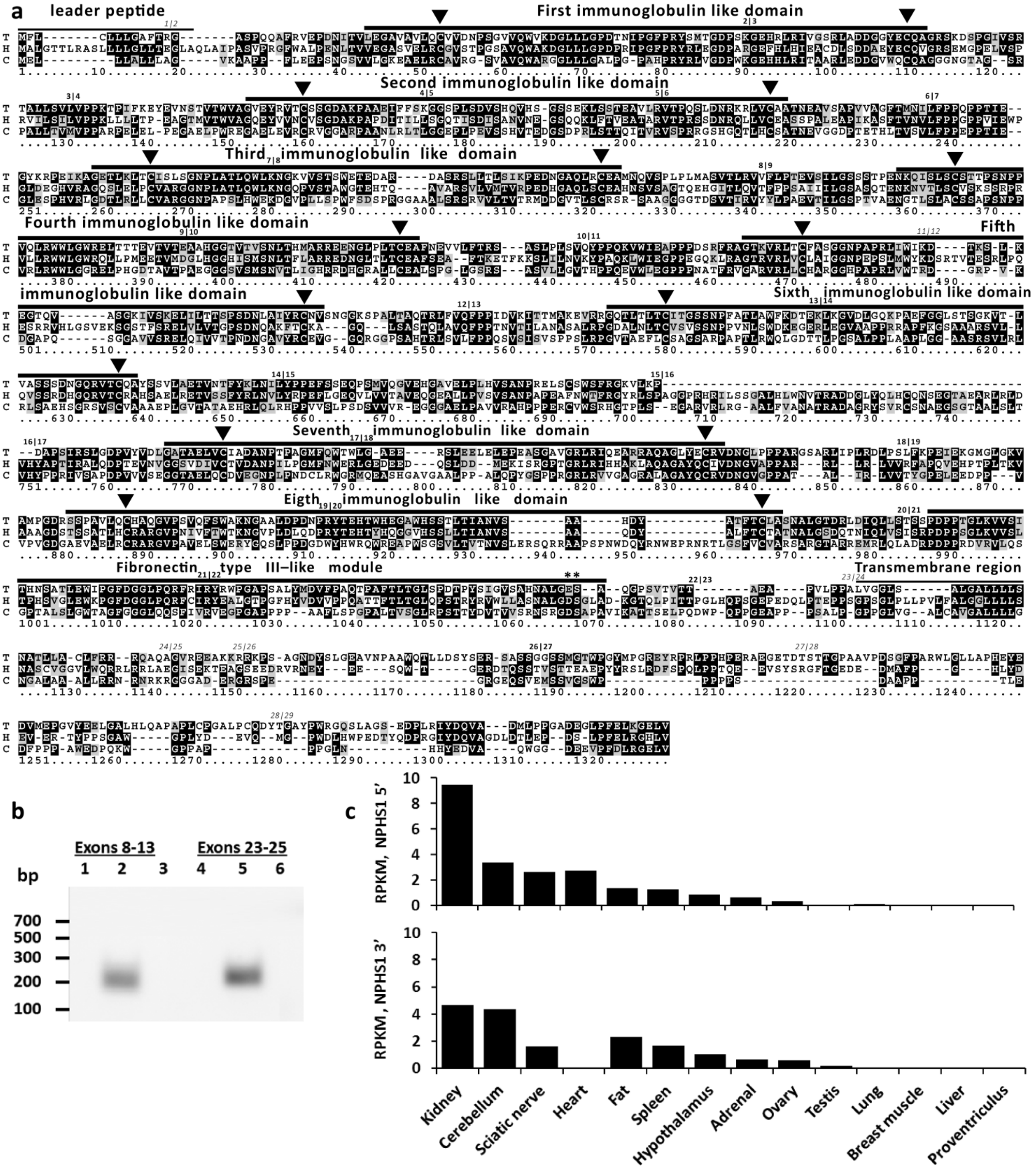

Figure S1: Predicted full length cDNA sequence of NPHS1 and its characterization. a Comparison of the predicted amino-acid sequence of *NPHS1* from human (H, GenBank accession no. NP_004637), green turtle (T, GenBank accession no. XP_007066218.1) and chicken (C, our submission). Dashes indicate gaps in the alignment. Identical, similar, and non-conserved residues are indicated by a black, grey, and white background, respectively. The signal peptide and the structural elements including eight Ig-like domains, a fibronectin type III–like module and a transmembrane domain of the human NPHS1 (Kestila M, Lenkkeri U, Mannikko M, Lamerdin J, McCready P, Putaala H, Ruotsalainen V, Morita T, Nissinen M, Herva R, et al: **Positionally cloned gene for a novel glomerular protein nephrin is mutated in congenital nephrotic syndrome.** *Mol Cell* 1998, **1:**575-582) are denoted above the alignment. The two conserved cysteines that mediate the folding of each of the Ig-like domains are indicated by black arrowheads. Two asterisks indicate the two residues (DS) that form the cytokine receptor motif in the fibronectin module. Exon borders of the human gene are indicated by the exon numbers in bold (conserved between species) or italics (non-conserved). **b** RT-PCR amplification of the 5' and 3' regions of chicken *NPHS1*. Both regions were amplified by two-step PCR: the first PCR included exons 8 to 13 and 23 to 25, with expected product sizes of 722 and 561 bp, respectively (lanes 1 and 4). Nested PCR for each of the products included exons 9-11 (207 bp) and exons 23-24 (249 bp), respectively (lanes 2 and 5). Primer sequences are listed in Additional file 2: Table S11. Sequence analysis of the amplified fragment confirmed the predicted mRNA sequence. **c** Chicken *NPHS1* expression profile based on RNA-Seq data from adult red junglefowl (GenBank accession number ERA252218), using as baits two non-overlapping probes of the predicted *NPHS1* cDNA, corresponding to the conserved extracellular domain (upper panel) and the non-conserved intracellular domain (lower panel), which are listed in Additional file 2: Table S12. Data were calculated as reads per kilobase per million mapped reads (RPKM).

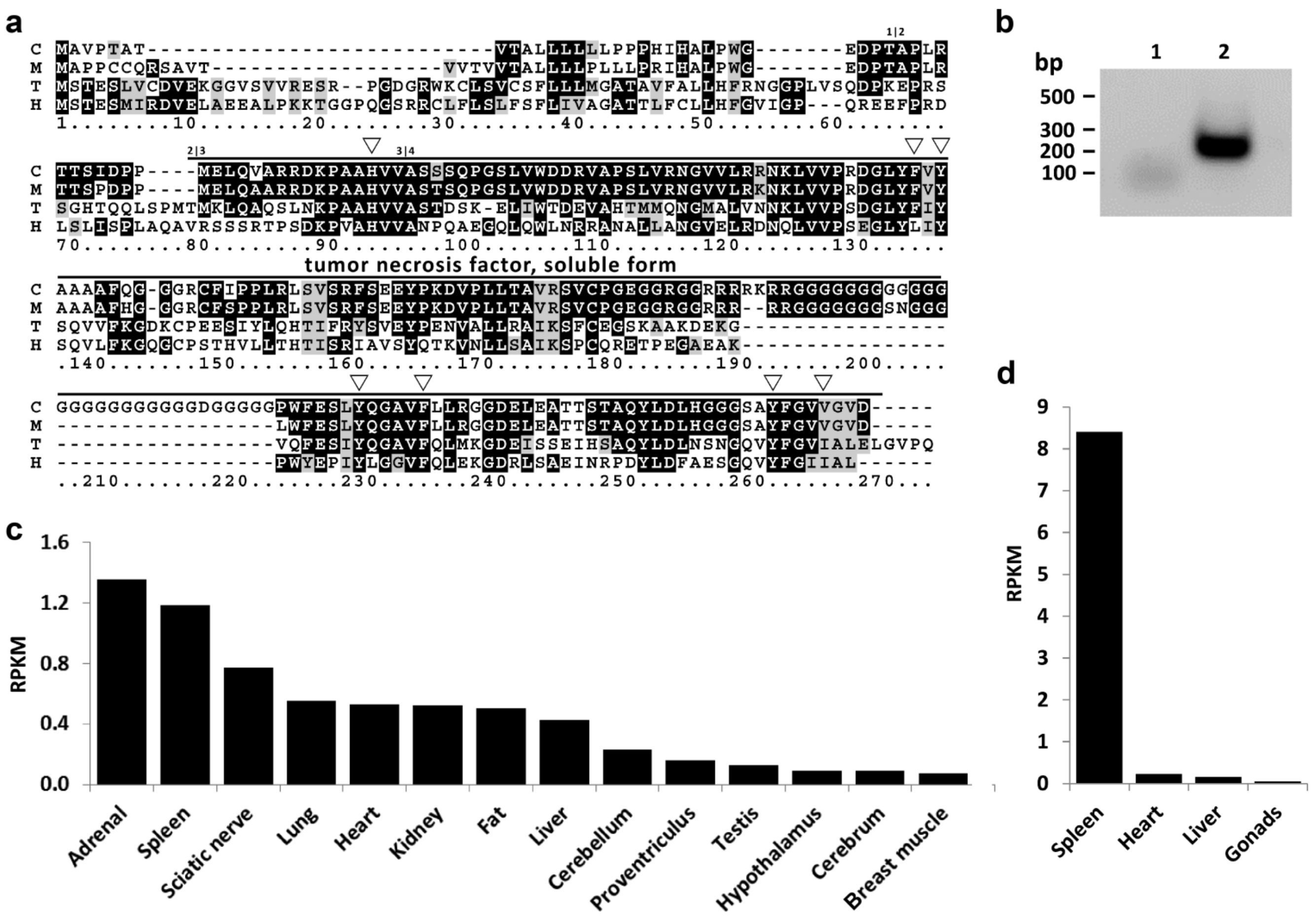

Figure S2: Predicted full length cDNA sequence of TNF and its characterization. a Comparison of predicted amino-acid sequence of TNF from green turtle (T, GenBank accession no. XP_007053265.1), human (H, GenBank accession no. NP_000585.2), and chicken (C, Additional file 2: Table S9). Dashes indicate gaps in the alignment. Identical, similar, and non-conserved residues are indicated by a black, grey, and white background, respectively. The positions of the four exon borders are indicated above the text. **b** RT-PCR amplification of the predicted *TNF* by two step PCR using chick spleen RNA as template. Amplification with the first set of primers did not yield a detectable signal on the ethidium-bromide gel (lane 1). An expected size band was evident with nested primers (lane 2) and was confirmed by sequencing. Primer sequences are listed in Additional file 2: Table S11. **c** Expression profile of chicken *TNF* in adult red junglefowl tissues based on RNA-Seq data as described in Additional File 2: Table S1c **d** RNA-Seq data from 19-days chick-embryos of Leghorn type (SRA269497). Bait sequence of *TNF* included 340 bp of the 5’ end of the transcript (Additional file 2: Table S12).

Table S9: Coding sequence of chicken *NPHS1* and *TNF* predicted transcripts.

*NPHS1**

ATGGAGCTGCTGTTGGCACTGCTGTTGGCCGGGGTCAAAGCGGCCCCGCCCTTCCTGGAGGAGCCGTCCAATGGCAGCGTGGTGTTGGGGA
AGGAGGCGGAGCTCCGCTGCGCCGTGCGGGGGAGCGTGGCTGTGCAGTGGGCACGGGGGGGGCTGCTGCTGGGGGCGCTGCCAGGCCCCGC
CCACCCGCGATACCGATTGGTCGGCGACCCGTGGAAAGGAGAGCACCACCTGCGCATCACGGCCGCGCGATTGGAGGACGACGGCGTCTGG
CAGTGCCAGGCGGGGGGGGGGAACGGAACTGCGGGGTCACGGCCTGCACTGCTAACGGTGATGGTGCCCCCCGCCCGCCCAGAGCTGGAGC
TGCCCGAGGGGGCGGAGCTGCCGTGGCGGGAGGGGGCGGAGCTGGAGGTGCGGTGCCGAGTGGGCGGGGCAAGGCCGGCGGCCAATCTGCG
GCTGACATTGGGGGGGGAGCCGCTGCCGGAGGTCTCCTCCCACGTCACTGAGGATGGCAGCGACCCCCGGCTGAGCACCACGCAGATCACC
GTCCGTGTGTCCCCCCGCCGCGGCTCCCACGGGCAGACCCTGCACTGTTCGGCCACCAATGAGGTGGGGGGGGACCCCACGGAGACCCACC
TGACGGTCAGCGTGCTCTTTCCCCCCGAGCCGCCCACCATCGAGGGTTTGGAGTCGCCCCACGTGAGGTTGGGGGACACTCTGCGACTGCT
CTGCGTGGCACGGGGGGGCAACCCGGCCCCCAGTCTGCACTGGGAGAAGGACGGCGTCCCGCTGCTCTCTCCGTGGTTCTCCGACTCGCCG
CGTGGGGGCGCTGCGGCGCTGTCCCGCAGCCGGGTGGTGCTGACGGTGACGCGGATGGACGACGGCGTCACTCTGAGCTGCCGCAGCCGCA
GCGCCGCGGGGGGGGGCGGCACCGACAGCGTCACCATCCGCGTGTACTACCTTCCGGCGGAGGTGACGATTTTGGGGTCTCCGACGGTGGC
GGAGAACGGGACGCTGAGCCTGGCGTGCAGCAGCGCCCCCAGCAACCCCCCGGTGCGGCTGCGCTGGTGGCTGGGGGGGCGCGAGCTGCCC
CACGGGGACACCGCCGTCACACCGGCCGAGGGCGGCGGTTCCGTCTCGATGTCCAACGTGACGCTGATTGGGCACCGCCGCGACCACGGGA
GGGCGCTGCTGTGCGAGGCGCTGAGCCCGGGGCTGGGGAGCCGCAGTGCCAGCGTCCTGCTGGGGGTCACACACCCCCCGCAGGAGGTGTG
GCTCGAGGGTCCCCCCCCCAACGCCACCTTCCGGGTGGGCGCCCGCGTCCGGCTGCTGTGCCACGCCCGGGGGGGGCACCCCGCGCCCCGC
CTCGTCTGGACCAGGGACGGCCGCCCCGTTAAGGACGGCGCCCCTCAGTCTGGGGGTGCGGTGGTCTCGCGGGAGCTGCAGATCGTGGTGA
CCCCAAATGACAACGGGGCCGTATACCGCTGCGAGGTGGGGGGGCAGCGGGGGGGGCCTTCCGCCCATACACGGCTCAGCGTCCTCTTCCC
GCCGCAGTCGGTGTCCATCTCGGTGTCGCCGCCGTCGCTCCGTCCCGGCGTCACCGCGGAGTTCCTCTGCAGCGCCGGCAGCGCCCGCCCC
GCCCCCACCCTGCGATGGCAGCTCGGGGACACCACCCTCCCCGGCTCAGCGCTGCCTCCGTTGGCGGCGCCGTTGGGCGGCGCGGCGTCGC
GCTCAGTGCTGCGGCTGCGGCTGTCGGCGGAGCATTCGGGCCGCAGCGTTTCGTGCGTGGCCGCCGCGGAGCCGTTGGGGGTCACCGCCAC
CGCCGAACACCGCCTGCAGCTGCGCCACCCCCCCGTGGTGTCGCTGCCGTCGGACTCCGTGGTGGTGCGGGAGGGGGGCGGGGCGGAGCTG
CCGGCCGTCGTGCGCGCCCATCCCCCACCCGAGCGCTGCGTCTGGAGCCGCCACGGGACACCGCTCAGCGAGGGCGCCCGAGTGCGGCTGC
GCGGAGCGGCGCTGTTTGTGGCCAACGCGACGCGGGCGGACGCGGGGCGGTACAGCGTGCGCTGCAGCAACGCCGAAGGGAGCGGCACCGC
CGCGCTGAGCCTCACCGTGCACTACCCTCCCCGCATTGTGTCAGCTCCGGACCCGGTGGTGGTGTCGGAGGGCGGCACAGCGGAGCTGCAG
TGCGACGTGGAGGGGAACCCTCTGCCCAACGACTGCCTGCGCTGGGGGAGGATGCAGGAGGCGTCCCATGGGGCGGTGGGGGCGGCGCTGC
CCCCCGCGCTGCAGCCCTATGGTTCCCCCCCCCGGGGCCGCCTGCGGGTGGTGGGGGCGGGGCGGGCGCTGGCGGGCGCCTATCAGTGCCG
CGTGGACAACGGGGTGGGACCCCCCGCAACCGCACTCATCCGCCTCGTCGTCACCTACGGCCCGGAATTGGAGGAGGACCCTCCAGTGGTT
CCGGTTGGGGACGGCGCCGAAGTGGCGGAGCTGCGCTGCCGGGCTCGGGGGGTTCCGGCTGTGGAGCTCAGCTGGGAGCGCTACGGCCAAT
CACTGCCGCCTGATGGAGACTGGTACCACTGGCGGCAGTGGCGGGAGGCTCCGTGGAGCGGCAGCGTTCTGACGGTGACCAACGTCAGCCT
GGAGCGCTCCCAGCGCCGCGCCGCCCCCTCCCCAAACTGGGACCAGTACCGCAACTGGGAGCCCCGCAACCGCACCCTGGGCAGCTTCGTG
TGCGTGGCGCGCAGCGCCCGCGGCACCGCCAGGAGGGAGATGAGGCTGCAGCTGGCCGACCGGCCGGACCCCCCCCGCGATGTGCGCGTGT
TGCAGAGCGGCCCCACAGCGCTGAGCCTCGGATGGACGGCCGGATTTGGGGGGGGGCTGCAGCAGAGCTTCATTGTCAGGGTTGAGGGTCC
CGGCGCCCCGCCCCCCCCCGCCGCGTTCCTCTCTCCGGGCCCCGCCCTCACCGTGAGCGGCCTCCGCCCGTCCACCACTTATGACGTCACC
GTCGTCTCACGCAACTCCCGGGGGGACAGCGCCCCCGCGGTCATCAAGGCCACCACTTCCGAGCTGCCCCAGGATTGGCCGCAGCCCCCCG
GCGAAGCCCCGCCCCCTTCCGCTCTGCCCGGTCCTCCCGGCCTGGTGGGGGCGCTGTGCGCTGTGGGGGCGCTGCTGCTGCTGGGGAACGG
AGCGCTGGCAGCGGCCCTACTGAGGCGGAACCGGAACCGGAAGCGGGGGGGCGGGGCCGACGAAAGGGGGCGGAGCCCGGAAGGGCGGGGA
GAGCAGAGTGTGGAGATGAGCTCTGTGGGATCGTGGCCGCCCCCCCCCCCCAGCGACGCCGCCCCCCCCACCTTGGAGGACTTCCCCCCCC
CCGCCTGGGAGGACCCCCAAAAATGGGGTCCCCCCGCGCCCCCCCCGGGGCTGAACCATCACTACGAGGACGTGGCCCAATGGGGGGGGGA
CGAAGAGGTGCCCTTTGACCTCCGAGGGGAGCTGGTGTGA

*TNF**

ATGGCCGTCCCCACCGCCACCGTCACCGCTCTGCTGCTGCTGCTGCTGCCGCCGCCCCACATCCACGCGCTGCCGTGGGGAGAGGACCCCA
CAGCTCCGCTCAGAACGACGTCAATTGACCCCCCCATGGAGCTGCAGGTGGCGCGGAGGGACAAACCCGCAGCGCACGTCGTGGCATCGTC
CTCTCAGCCCGGCTCGTTGGTGTGGGACGACCGCGTTGCCCCATCCCTGGTCCGTAACGGAGTGGTGCTGAGAAGGAACAAATTGGTGGTC
CCCCGGGACGGCCTTTACTTCGTATACGCCGCCGCCGCTTTCCAGGGGGGGGGGGCGTTGTTTCATCCCCCCCCCTGAGGCTGTCGGTCAGCC
GCTTCTCCGAGGAGTACCCCAAAGACGTCCCCCTCCTGACCGCCGTGCGCTCCGTCTGCCCCGGGGAAGGGGGGAGGGGGGGGAGGAGGAG
GAGGAAGAGGAGGGGGCGGGGGGGGGGGGGGGGGGGGGGGGGGGGGGGGGGGGGGGGGGGGGGGGGGGGGGGGGGGGGGGGGGACGGCGGT
GGGGGGGGGCCGTGGTTCGAGTCGCTGTATCAGGGGGCGGTGTTTTTGCTGAGGGGGGGGGGACGAGTTGGAGGCGACCACCAGCACGGCGC
AGTACCTCGACCTGCACGGGGGGGGGCAGCGCCTATTTCGGGGTGGTGGGAGTGGATTAA

*Initiation and stop codons are underlined

Table S10: List of *NPHS1* and *TNF* exons in human, turtle and chicken.

a *NPHS1*

	Human		Turtle		Chicken
1	MALGTTLRASLLLLGLLTE	2	(FATIHRGPSLSSLPVPAALVGMVSKGPVT)MFLCLLLGAFTR	1	ME
				2	LLLALLLAGVK
2	GLAQLAIPASVPRGFWALPENLTVVEGASVELRCGVSTPGSAVQWAKDGLLLGPDPRIPGFP<br>RYRLEGDPAR	3	GASPQQAFRVEPDNITVLEGAVAVLQCVVDNPSGVVQWVKDGLLLGPDTNIPGFPRYSMTGDPSK	3	AAPPFLEEPSNGSVVLGKEAELRCAVRGSVAVQWARGGLLLGALPGPAHPRYRLVGDPWK
3	GEFHLHIEACDLSDDAEYECQVGRSEMGPELVSPRVILSIL	4	GEHRLRIVGSRLADDGGYECQAGRSKDSPGIVSRTALLSVL	4	GEHHLRITAARLEDDGVWQCQAGGGNGTAGSRPALLTVM
4	VPPKLLLLTPEAGTMVTWVAGQEYVVNCVSGDAKPAPDITILL	5	VPPKTPIFKEYEVNSTVTWVAGVEYRVTCSSGDAKPAAEIFFSK	5	VPPARPELELPEGAELPWREGAELEVRCRVGGARPAANLRLTL
5	SGQTISDISANVNEGSQQKLFTVEATAR	6	GGSPLSDVSHQVHSGSSEKLSSTEAVLR	6	GGEPLPEVSSHVTEDGSDPRLSTTQITVR
6	VTPRSSDNRQLLVCEASSPALEAPIKASFTVNVL	7	VTPQSLDNRKRLVCAATNEAVSAPVVAGFTMNIL	7	VSPRRGSHGQTLHCSATNEVGGDPTETHLTVSVL
7	FPPGPPVIEWPGLDEGHVRAGQSLELPCVARGGNPLATLQWLK	8	FPPQPPTIEGYKRPEIKAGETLKLTCISLSGNPLATLQWLK	8	FPPEPPTIEGLESPHVRLGDTLRLLCVARGGNPAPSLHWEK
8	NGQPVSTAWGTEHTQAVARSVLVMTVRPEDHGAQLSCEAHNSVSAGTQEHGITLQVT	9	NGKVVSTSWETEDARDASRSLLTLSIKPEDNGAQLRCEAMNQVSPLPLMASVTLRVV	9	DGVPLLSPWFSDSPRGGAAALSRSRVVLTVTRMDDGVTLSCRSRSAAGGGGTDSVTIRVY
9	FPPSAIIILGSASQTENKNVTLSCVSKSSRPRVLLRWWLGWRQLLPMEETVMD	10	FLPTEVSILGSSSTPENKQISLSCSTTPSNPPVQLRWWLGWRELTTTEVTVTE	10	YLPAEVTILGSPTVAENGTLSLACSSAPSNPPVRLRWWLGGRELPHGDT
10	GLHGGHISMSNLTFLARREDNGLTLTCEAFSEAFTKETFKKSLILNVK	11	AAHGGTVTVSNLTHMARREENGLPLTCEAFNEVVLFTRSASLPLSVQ	11	AVTPAEGGGSVSMSNVTLIGHRRDHGRALLCEALSPGLGSRSASVLLGVT
11	YPAQKLWIEGPPEGQKLRAGTRVRLVCLAIGGNPEPSLMWYK	12	YPPQKVWIEAPPPDSRFRAGTKVRLTCFASGGNPAPRLIWIK	12	HPPQEVWLEGPPPNATFRVGAR
12	DSRTVTESRLPQESRRVHLGSVEKSGSTFSRELVLVTGPSDNQAKFTCKAGQLSASTQLAVQ	13	DTKSLKEGTQVASGKIVSKELILTTSPSDNLAIYRCNVSNGGKSPALTAQTRLFVQ	13	VRLLCHARGGHPAPRLVWTRDGRPVKDGAPQSGGAVVSRELQIVVTPNDNGAVYRCEVGGQRGGPSAHTRLSVL
13	FPPTNVTILANASALRPGDALNLTCVSVSSNPPVNLSWDKEGER	14	FPPIDVKITTMAKEVRRGQTLTLTCITGSSNPFATLAWFKDTEK	14	FPPQSVSISVSPPSLRPGVTAEFLCSAGSARPAPTLRWQLGDTT
14	LEGVAAPPRRAPFKGSAAARSVLLQVSSRDHGQRVTCRAHSAELRETVSSFYRLNVL	15	LKGVDLGQKPAEFGGLSTSGKVTLVASSSDNGQRVTCQAYSSVLAETVNTFYKLNIL	15	LPGSALPPLAAPLGGAASRSVLRLRLSAEHSGRSVSCVAAAEPLGVTATAEHRLQLR
15	YRPEFLGEQVLVVTAVEQGEALLPVSVSANPAPEAFNWTFRGYRLSP	16	YPPEFSSEQPSMVQGVEHGAVELPLHVSANPRELSCSWSFRGKVLKP	16	HPPVVSLPSDSVVVREGGGAELPAVVRAHPPPERCVWSRHGTPLSG
16	AGGPRHRILSSGALHLWNVTRADDGLYQLHCQNSEGTAEARLRLDVH			17	EGARVRLRGAALFVANATRADAGRYSVRCSNAEGSGTAALSLTVH
17	YAPTIRALQDPTEVNVGGSVDIVCTVDANPILPGMFNWERL	17	DAPSIRSLGDPVYVDLGATAELVCIADANPTPAGMFQWTWL	18	YPPRIVSAPDPVVVSEGGTAELQCDVEGNPLPNDCLRWGRM
18	GEDEEDQSLDDMEKISRGPTGRLRIHHAKLAQAGAYQCIVDNGVAPPARRLLRLVVR	18	GAEERSLEELELEPEASGAVGRLRIQEARRAQAGLYECRVDNGLPPPARGSARLIPLRDLPSL	19	QEASHGAVGAALPPALQPYGSPPRGRLRVVGAGRALAGAYQCRVDNGVGPPATALIRLVVT
19	FAPQVEHPTPLTKVAAAGDSTSSATLHCRARGVPNIVFTWTKNGVPLDLQDPR	19	FKPEIEKGMGLGKVAMPGDRSSPAVLQCHAQGVPSVQFSWAKNGAALDPDNPR	20	YGPELEEDPPVVPVGDGAEVAELRCRARGVPAVELSWERYGQSLPPDGDW
20	YTEHTYHQGGVHSSLLTIANVSAAQDYALFTCTATNALGSDQTNIQLVSI	20	YTEHTWHEGAWHSSTLTIANVSAAHDYATFTCLASNALGTDRLDIQLLST	21	YHWRQWREAPWSGSVLTVTNVSLERSQRRAAPSPNWDQYRNWEPRNRTLGSFVCVARSARGTARREMRLQLA
21	SRPDPPSGLKVVSLTPHSVGLEWKPGFDGGLPQRFCIR	21	SSPDPPTGLKVVSITHNSATLEWIPGFDGGLPQRFRIR	22	DRPDPPRDVRVLQSGPTALSLGWTAGFGGGLQQSFIVR
22	YEALGTPGFHYVDVVPPQATTFTLTGLQPSTRYRVWLLASNALGDSGLADKGTQLPITTP	22	YRWPGAPSALYMDVFPAQTPAFTLTGLSPDTPYSIGVSAHNALGESAQGPSVTVTTA	23	VEGPGAPPPPAAFLSPGPALTVSGLRPSTTYDVTVVSRNSRGDSAPAVIKATTSELPQDWPQPPGEAPP
23	GLHQPSGEPEDQLPTEPPS				
24	GPSGLPLLPVLFALGGLLLLSNASCVGGVLWQRRLRRLAE	23	EAPVLPPALVGGLSALGALLLLSNATLLACLFRRRQAQA		
25	GISEKTEAG	24	GVREEAKKR		
26	SEEDRVRNEYEESQWTGERDTQSST	25	RKPSAGNDYSLGEAVNPAAWQTLLDSYSERSASSG	24	PSALPGPPGLVGALCAVGALLLLGNGALAAALLRRNRNRKRGGGADERGRSPEGRGEQSVE
27	VSTTEAEPYYRSLRDFSPQLPPTQEEVSYSR	26	GSSMGTWPGYMPGREYRPRLPPHPERAEGETDTSTT		
28	GFTGEDEDMAFPGHLYDEVERTYPPSGAWGPLYDEVQM	27	GPAAVPDSGFPARWLGLLAPHEYEDVMEPGVYEELGALHLQAPAPLCPGALPCQDYT	25	MSSVGSWPPPPPSDAAPPTLEDFPPPAWEDPQKWGPPAPPPGLNHHYEDVAQWGGD
29	GPWDLHWPEDTYQDPRGIYDQVAGDLDTLEPDSLPFELRGHLV*	28	GAYPWRGQSLAGSEDPLRIYDQVADMLPPGADEGLPFELKGELV*	26	EEVPFDLRGELV*

b *TNF*

	Human		Turtle		Chicken
1	MSTESMIRDVELAEEALPKKTGGPQGSRRCLFLSLFSFLIVAGATTLFCLLHFGVIGPQREE	1	MSTESLVCDVEKGGVSVVRESRPGDGRWKCLSVCSFLLLMGATAVFALLHFRNGGPLVSQ	1	MAVPTATVTALLLLLLPPPHIHALPWGE
2	FPRDLSLISPLAQAV	2	DPKEPRSSGHTQQL	2	DPTAPLRTTSI
3	RSSSRTPSDKPVAHVV	3	SPMTMKLQAQSLNKPAAHVV	3	DPPMELQVARRDKPAAHVV
4	ANPQAEGQLQWLNRRANALLANGVELRDNQLVVPSEGLYLIYSQVLFKGQGCPSTHVLLTHTI<br>SRIAVSYQTKVNLLSAIKSPCQRETPEGAEAKPWYEPIYLGGVFQLEKGDRLSAEINRPDYLD<br>FAESGQVYFGIIAL	4	ASTDSKELIWTDEVAHTMMQNGMALVNNKLVVPSDGLYFIYSQVVFKGDKCPEESIYLQHTIFRYSV<br>EYPENVALLRAIKSFCEGSKAAKDEKGVQFESIYQGAVFQLMKGDEISSEIHSAQYLDLNSNGQVYF<br>GVIALELGVPQ*	4	ASSSQPGSLVWDDRVAPSLVRNGVVLRRNKLVVPRDGLYFVYAAAAFQGGGRCFIPPLRLSVSRFSEEYPKDVPL<br>LTAVRSVCPGEGGRGGRRRRKRRGGGGGGGGGGGGGGGGGGGGGDGGGGGPWFESLYQGAVFLLRGGDELEAT<br>TSTAQYLDLHGGGSAYFGVVGVD*

Table S11: List of primers used for RT-PCR.

Gene Name	Primers' Sequence (5' to 3')		Amplicon
	Forward	Reverse	(bp's)
TNF			
First	CGTAACGGAGTGGTGCTGAG	CCCTGATACAGCGACTCGAA	347
Nested	AACAAATTGGTGGTCCCCCG	TCCGTCTGCCCCGGGGAA	174
NPHS1			
N'-First	CAGTCTGCACTGGGAGAAGG	ATACGGCCCCGTTGTCATTT	722
N'-Nested	ATCCGCGTGTACTACCTTCC	TCACGTTGGACATCGAGACG	208
C'-First	GCAGAGCTTCATTGTCAGGG	GCCACGTCCTCGTAGTGATG	561
C'-Nested	GTCATCAAGGCCACCACTTC	CTCCACACTCTGCTCTCCC	249

N' , C' = N-terminal and C-terminal sets of primers

Table S12: Probes used for expression profiling in the Sequence Read Archive (SRA) database.

NPHS1 5'

CTCAGTTTGGCTGCTGAGAGCGGCATGGAGCTGCTGTTGGCACTGCTGTTGGCCGGGGTCAAAGCGGCCCCGCCCTTCCTGGAGGAGCCGTCCAATGGCAGCGTGGTGTT
GGGGAAGGAGGCGGAGCTCCGCTGCGCCGTGCGAGGGGGCGTGGCCGTGCAGTGGGCGCGGGGGGGGCTGCTGCTGGGGGCGCTGCCAGGCCCCGCCCACCCGCGATACC
GATTGGTCGGCGACCCGCGGAAAGGAGAGCACCACCTGCGCATCACGGCCGCGCGATTGGAGGACGACGGCGTCTGGCAGTGCCAGGCGGGGGGGGGGAACGGAACTGCG
GGGTCACGGCCTGCACTGCTAACGGTGATGGTGCCCCCCGCCCGCCCAGAGCTGGAGCTGCCCGAGGGGGCGGAGCTGCCGTGGCGGGAGGGGGCGGAGCTGGAGGTGCG
GTGCCGAGTGGGCGGGGCCAGGCCGGCGGCCAATCTGCGGCTGACGTTGGGGGGGGAGCCGCTGCCGGAGGTCTCCTCCCACGTCACTGAGGATGGCAGCGACCCCCGGC
TGAGCACCACGCAGATCACCGTCCGTGTGTCCCCCCGCCGCGGTTCCCACGGGCAGACCCTGCACTGTTCGGCCACCAATGAGGCGGGGGGGGACCCCACGGAGACCCAC
CTGACGGTCAGCGTGCTCTTTCCCCCCGAGCCGCCCACCATCGAGGGTTTGGAGTCGCCCCACGTGAGGTTGGGGGACACTCTGCGACTGCTCTGCGTGGCACGGGGGGG
CAACCCGGCCCCCAGTCTGCACTGGGAGAAGGACGGCGTCCCGCTGCTCTCTCCGTGGTTCTCCGACTCGCCGCGTGGGGGCGCTGCGGCGCTGTCCCGCAGCCGGGTGG
TGCTGACGGTGACGCGGATGGACGACGGCGTCACTCTGAGCTGCCGCAGCCGCAGCGCCGCGGGGGGGGGCGGCACCGACAGCGTCACCATCCGCGTGTACTACCTTCCG
GCGGAGGTGACGATTTTGGGGTCTCCGACGGTGGCGGAGAACGGGACGCTGAGCCTGGCGTGCAGCAGCGCCCCCAGCAACCCCCCGGTGCGGCTGCGCTGGTGGCTGGG
GGGGCGCGAGCTGCCCCACGGGGACACCGCCGTCACACCGGTGGGCAACCGCGGCACCCCGCCACCCCCACGCAGGACCCCACTGCGGCCCCACGGGGACCCATTGTGTC
CCATTGTGT

NPHS1 3' probe (264 bp)

GGCGGAGCCCGGAAGGGCGGGGAGAGCAGAGTGTGGAGATGAGCTCTGTGGGATCGTGGCCGCCCCCCCCCCCCAGCGACGCCGCCCCCCCCACCTTGGAGGACTTCCCC
CCCCCCGCCTGGGAGGACCCCCAAAAATGGGGTCCCCCGCGCCCCCCCCGGGGCTGAACCATCACTACGAGGACGTGGCCCAATGGGGGGGGGACGAAGAGGTGCCCTT
TGACCTCCGAGGGGAGCTGGTGTGACCCCCCCCCCCCAATAAAC

TNF

ATGGCCGTCCCCACCGCCACCGTCACCGCTCTGCTGCTGCTGCTGCTGCCGCCGCCCCACATCCACGCGCTGCCGTGGGGAGAGGACCCCACAGCTCCGCTCAGAACGAC
GTCAATTGACCCCCCCATGGAGCTGCAGGTGGCGCGGAGGGACAAACCCGCAGCGCACGTCGTGGCATCGTCCTCTCAGCCCGGCTCGTTGGTGTGGGACGACCGCGTTG
CCCCATCCCTGGTCCGTAACGGAGTGGTGCTGAGAAGGAACAAATTGGTGGTCCCCCGGGACGGCCTTTACTTCGTATACTTCGAGTCGCTGTATCAGGGGGCGGTGTTT
TTGCTGAGG
