# Supplementary material for: Correspondence on Lovell et al.: identification of chicken genes previously assumed to be evolutionarily lost
Source: Genome Biol. 2017 Jun 14;18:112. doi: 10.1186/s13059-017-1231-1 (PMC5470226; doi:10.1186/s13059-017-1231-1)
Supplement: Supplementary file 3 — Characterization of the high confidence novel genes. Table S13. Phylogenetic analysis of representative novel genes. Figure S3. Characterization of the novel transcripts. (PDF 232 kb) [file 13059_2017_1231_MOESM3_ESM.pdf]

Additional file 3: Characterization of the high confidence novel genes

Table S13: Phylogenetic analysis of representative novel genes.

Species	EV[1]	*ABHD14B*	*ATP5SL*	*B4GAT1*	*B9D2*	*CACNG7*	*COPZ1*	*ETFB*	*NAA10*	*OPA3*	*RPS19*	*RPS9*	*SLC50A1*	*SNRPD2*	*TXNIP*	*ZNRD1*
		Amino acid identity (%)														
C. japonica[2]	40															
A. platyrhynch[3]	84												51.3		91.7	
H. leucocephalus[4]	102		66.1		81.4		98.0						57.1	100.0	91.5	57.1
F. cherrug[5]	102	80.5				94.8	89.5						58.5		89.1	
T. guttata[6]	102			67.0				71.5				99.5				
C. livia[7]	102	78.6					96.3								89.9	
C. mydas[8]	253	71.0	60.7				97.1						56.3		82.4	
A. carolinensi[9]	278	68.6	45.0		71.6	84.3	95.5		82.6	82.5		98.9	55.3	100.0		47.1
M. musculus[10]	321	64.3	42.1	56.9	69.9	89.5	96.6	66.0		74.8	95.9	99.4	50.3	100.0	78.5	49.6
H. sapiens[11]	321	64.5	45.7	56.2	69.9	89.5	96.6	66.0	82.5	75.7	95.9	99.4	48.0	100.0	78.6	48.7
X. laevis[12]	356	60.4		60.0	65.3		91.5	64.1	81.9	76.3	91.0	97.8	52.2	98.3	74.7	
D. rerio[13]	430	58.0		51.5	68.6		92.1	63.2	73.1	76.3	87.6	95.6	47.8	99.2	62.5	38.8
Correlation (aa identity *vs*. EV)		-1.0	-0.9	-0.9	-0.9	-0.7	-0.2	-1.0	-0.9	-0.5	-1.0	-0.7	-0.7	-0.5	-1.0	-0.9
GC-content (%)[14]		70.5	66.9	69.5	70.6	66.9	63.2	69.1	60.0	68.1	65.3	68.3	70.4	62.0	67.0	61.5

[1]Evolutionary distance from chicken in million years (MY), of the listed vertebrates was estimated using TimeTree web page: http://www.timetree.org [1]. [2]*Coturnix japonica* (Japanese quail);[3]*Anas platyrhynchos* (mallard), [4]*Haliaeetus leucocephalus* (Bald eagle); [5]*Falco cherrug* (Saker falcon); [6]*Taeniopygia guttata* (Zebra finch); [7]*Columba livia* (Rock dove); [8]*Chelonia mydas* (Green sea-turtle);[9]*Anolis carolinensis* (Green anole); [10]*Mus musculus* (Mouse); [11] *Homo sapiens* (Human); [12]*Xenopus laevis* (African clawed frog); [13]*Danio rerio* (Zebrafish). [14]GC-content in chicken.

One trinity transcript (*MRPL11*) was excluded from this study due to inclusion of an intron in *Chelonia mydas* [Accession no. XR_001463072], leading to a reduced similarity score.

1. Hedges SB, Marin J, Suleski M, Paymer M, Kumar S: **Tree of life reveals clock-like speciation and diversification.** Mol Biol Evol 2015, 32:835-845.

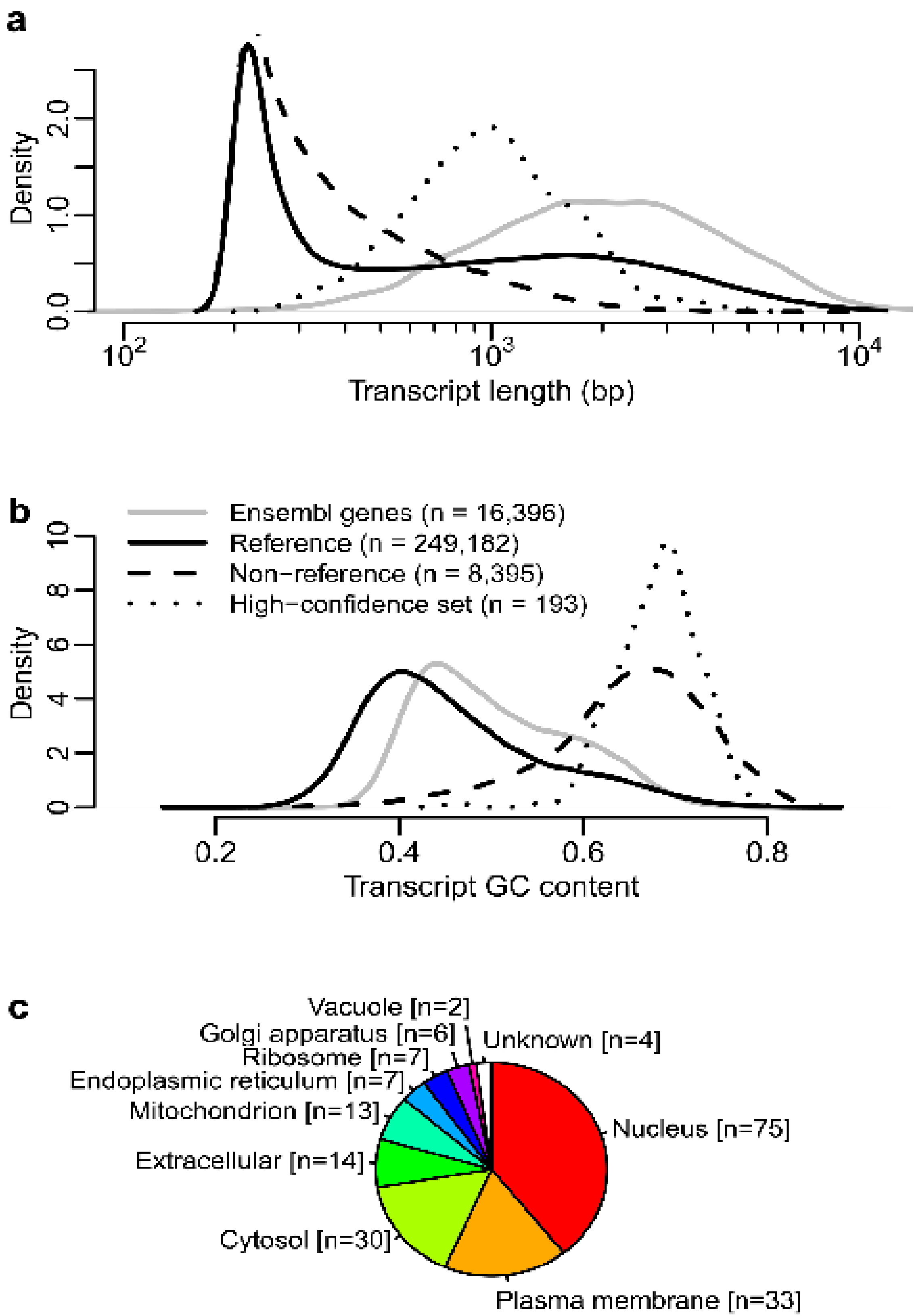

Figure S3: Characterization of the novel transcripts. a Length distribution of the Trinity

transcripts compared to protein-coding genes annotated in Ensembl. The ‘reference’ and ‘non-reference’ sets represent Trinity transcripts with or without alignment to the reference genome, respectively, and the ‘high-confidence set’ is the final set of novel genes selected from the non-reference set. **b** Distribution of GC-content in the Trinity transcripts compared to the protein-coding genes annotated in Ensembl as described in a. **c** Pie chart of the high confidence novel genes based on predicted subcellular localization. This prediction was made according to the mammalian orthologs using PANTHER, MGI and GOrilla software. The specific genes in each category are listed in Additional file 1: Table S7.

1. Hedges SB, Marin J, Suleski M, Paymer M, Kumar S: **Tree of life reveals clock-like speciation and diversification.** *Mol Biol Evol* 2015, **32:**835-845.
