# Supplementary material for: Correspondence on Lovell et al.: identification of chicken genes previously assumed to be evolutionarily lost
Source: Genome Biol. 2017 Jun 14;18:112. doi: 10.1186/s13059-017-1231-1 (PMC5470226; doi:10.1186/s13059-017-1231-1)
Supplement: Supplementary file 4 — Detailed materials and methods. Animals and tissue sampling. RNA-seq. Bioinformatic analysis. RT-PCR. Mass spectrometry analysis (MS). (PDF 283 kb) [file 13059_2017_1231_MOESM4_ESM.pdf]

# Additional file 4: Detailed Materials and Methods

## Animals and tissue sampling

Female broiler (Cobb) and White Leghorn (Lohman) chickens were purchased from commercial husbandries (Brown & Sons and Hasolelim, Israel, respectively) at the age of 1 day and grown according to recommended husbandry and feeding conditions (NRC 1994) with free access to food and water. At sexual maturation, as indicated by egg lay (about 4 month of age), samples of abdominal (visceral) fat, hypothalamus, and pituitary were snap-frozen in liquid nitrogen after neck dislocation. Total RNA was prepared using a RNA isolation kit (miRNeasy, Qiagen).

## RNA-seq

Libraries of cDNA were prepared by the Uppsala sequencing platform from 1 μg RNA using the TruSeq Stranded mRNA Sample Preparation Kit (Illumina Inc, #15031047 Rev E). Libraries were prepared, from three individual birds of each chicken strain and three tissues (visceral fat, hypothalamus and pituitary). The libraries were uniquely tagged and sequenced with Illumina HiSeq, producing around 33 million paired-end reads (2x124 bp) per library.

## Bioinformatic analysis

Trim Galore! (http://www.bioinformatics.babraham.ac.uk/projects/trim_galore) and Trimmomatic (Bolger AM, Lohse M, Usadel B: **Trimmomatic: a flexible trimmer for Illumina sequence data.** *Bioinformatics* 2014, **30:**2114-2120) were used to remove adaptor sequences and low-quality base calls from the sequencing reads. Firstly, Trim Galore! 0.3.7 was used with the parameters "-q 15 -e 0.1 -O 6 --length 36", followed by Trimmomatic 0.32 with the parameters "SLIDINGWINDOW:10:30 LEADING:5 TRAILING:5 MINLEN:36" to remove larger regions of low-quality base calls. Resulting single-end reads were removed before further analysis. The quality of the resulting reads was verified using FASTQC (http://www.bioinformatics.babraham.ac.uk/projects/fastqc).

*De-novo* assembly was performed using Trinity r20140717 (Grabherr MG, Haas BJ, Yassour M, Levin JZ, Thompson DA, Amit I, Adiconis X, Fan L, Raychowdhury R, Zeng Q, et al: **Full-length transcriptome assembly from RNA-Seq data without a reference genome.** *Nat Biotechnol* 2011, **29:**644-652) with strand-specific RNA-Seq reads and *in silico* read normalization enabled. All 18 samples were merged for the assembly. Gene and transcript abundance was estimated with Trinity. Only transcripts with at least a total FPKM of 2 across all samples, or 1 in any one sample were kept. Next, we used both BLAT v. 35 and BLAST 2.2.29+ to map the transcripts to the Galgal4 reference genome in order to identify putative novel transcripts. BLAT was run with default parameters and BLAST was run with '-num_threads 4 -outfmt 6 -max_target_seqs 1'.

Functional annotation was done following the instructions for Trinotate v2.0. The SwissProt, Uniref90, and Pfam domains databases were downloaded from the Trinotate website. The TransDecoder tool was used to predict the coding sequence of each transcript based both on open reading frames and the resulting polypeptide score. The nucleotide and amino acid sequences were then blasted against SwissProt using BLAST 2.2.29+. In addition, the amino acid sequences were blasted against the more extensive UniRef database. Identification of protein domains in Pfam was performed using HMMER, signal peptides were predicted using signalP v4, and transmembrane domains were predicted using tmHMM v2. RNAMMER was used to identify and exclude rRNAs. The search for gene expression in the SRA database included the ChicksPress project of red junglefowl (http://geneatlas.arl.arizona.edu/sra_data.php).

Classification of the novel genes was performed using GeneCards (http://www.genecards.org/), PANTHER (http://pantherdb.org/), MGI (http://www.informatics.jax.org/genes.shtml) and PubMed. Enrichment analysis was performed using the GOrilla software (http://cbl-gorilla.cs.technion.ac.il/).

**RT-PCR**

Amplification of *NPHS1* and *TNF* cDNAs were done using mRNA from the kidneys of adult Leghorn chickens and spleen of 19-day embryos, respectively, and the My Taq HSRed kit (BioLine, Modi'in, israel). To adjust for the high GC-content of the transcripts DMSO (2.5%) and betain (0.125M) were added to the supplied reaction buffer. Cycling conditions were: initial denaturation at 95°C for 3 min; 30 cycles at 95°C for 1 min; annealing at 60°C to 55°C (always 2 °C below the melting point of primers) for 20 s; polymerization at 72°C for 1 min (3 min for the long templates of *NPHS1*); and final extension step at 72°C for 10 min. PCR products were separated on 2% agarose gels with ethidium bromide and visualized by UV. Size estimation of the PCR products was done using Quick-Load100 bp DNA Ladder (NEB, Ormat, Rehovot, Israel). Products were verified by sequencing. Primer sequences are listed in Additional file 2: Table S10.

**Mass spectrometry analysis (MS)**

Proteomics analysis was performed on roughly 50 mg visceral fat from each of three individuals of each chicken strain. Samples were washed with acetone to remove lipids and residual acetone was removed using vacuum centrifugation. Disulphide bonds were reduced using 5 µl 100 mM dithiothreitol, 100 mM ammonium bicarbonate (ABC) at 85°C for 5 min. Once returned to room temperature, reduced bonds were alkylated using 5 µl 100 mM iodoacetamide and 100 mM ABC for 30 min. Two µg proteomics grade trypsin (Sigma #T6567-20UG) dissolved in 100 µl 100 mM ABC was added to each sample and all were placed at 37°C overnight. Samples were desalted, after which residual lipids were removed using a strong cation exchange trap. A second desalting was necessary to remove high levels of salts from the strong cation exchange elution prior to mass spectrometry.

MS of tryptic peptides was performed using a Dionex U3000 splitless nano-flow HPLC system coupled with a Thermo LTQ Velos Pro linear ion trap mass spectrometer. Peptides were reverse-phase separated using an acetonitrile gradient from 2% to 50% in 4 h at a flow rate of 333 nl per min. Formic acid (0.1%) was used as a proton source. The C18 column, an in-house prepared 75 µm by 15 cm reverse phase column packed with Halo 2.7 µm, 90Å C18 material (MAC-MOD Analytical), was located in the ion source just before a silica emitter. A potential of 2100 volts was applied using a liquid junction between the column and emitter. A Thermo LTQ Velos Pro mass spectrometer using a nanospray Flex ion source was used to analyze the eluate from the U3000. Scan parameters for the LTQ Velos Pro were one MS scan followed by 10 MS/MS scans of the five most intense peaks. MS/MS scans were performed in pairs; a CID fragmentation scan followed a HCD fragmentation scan of the same precursor m/z. Dynamic exclusion was enabled with a mass exclusion time of 3 min and a repeat count of 1 within 30 s of initial m/z measurement. Raw mass spectra were converted to MGF text files using MSConvert, part of the ProteoWizard software library (PMID: 1860660). X!tandem 2013.09.01.1 (PMID: 14976030) and OMSSA 2.1.8 (PMID: 15473683) algorithms were employed via the University of Arizona High Performance Computing Center to perform spectrum matching. Precursor and fragment mass tolerance were set to 0.25 Daltons for both OMSSA and X!tandem. Trypsin cleavage rules were used for both algorithms with up to two missed cleavages. Amino acid modifications searched consisted of single and double oxidation of Methionine, oxidation of Proline, N-terminal acetylation, carbamidomethylation of Cysteine, deamidation of Asparagine and Glutamine and phosphorylation of Serine, Threonine, and Tyrosine. X!tandem xml and OMSSA xml results were compiled and filtered to remove any peptide matches with an E-value > 0.05 as well as proteins identified by a single peptide sequence. The protein fasta database used for spectrum matching was created from the Trinity *de novo* assembly using TransDecoder. A randomized version of the fasta was concatenated to the original as a way to assess data quality. The mass spectrometry proteomics data have been deposited to the ProteomeXchange Consortium (http://proteomecentral.proteomexchange.org) via the PRIDE partner repository (PMID:23203882) with the data set identifier.
